# We need to think about data governance for dementia research in a digital era

**DOI:** 10.1186/s13195-020-0584-y

**Published:** 2020-01-31

**Authors:** Richard Milne, Carol Brayne

**Affiliations:** 1 Society and Ethics Research Group, Wellcome Genome Campus, Hinxton, UK; 2 Institute of Public Health, University of Cambridge, Cambridge, UK

**Keywords:** Dementia, Ethics, Data governance, Data sharing, Data trust, Digital health

## Abstract

**Background:**

Research into Alzheimer’s disease and other dementias increasingly involves large-scale data-sharing initiatives. The development of novel digital tools and assessments is likely to increase the need for these. This presents ethics and governance challenges to ensure the use of these data is able to maximise the benefit to patients and the public.

**Discussion:**

We consider the challenges associated with informed consent and governance in the context of dementia research. We set out the potential of novel data governance approaches for the future of data sharing for dementia.

**Summary:**

The data trust model proposed in discussions of data governance may have potentially valuable application for dementia research. Such inclusive approaches to trustworthy data governance should be considered as data-sharing initiatives are established and develop.

## Background

Big data derived from clinical records and research studies or produced incidentally in everyday life represent an opportunity for dementia research, diagnosis and care [1, 2]. The application of machine learning and artificial intelligence techniques to cognitive, behavioural or biological data may contribute to the detection of early cognitive decline, improve our ability to model the course of the condition and help identify individuals who may be most suitable for clinical trials.

The scale and nature of data present both a challenge and an opportunity for the governance of data for dementia research. Challenges include maintaining the trust of members of the public and patients in the collection and use of data, ensuring that informed consent protects the interests of research participants and considering how to enable individuals to contribute their clinical or other data to research, and vice versa. There is also a continuing need to support researchers and companies to engage with data sharing.

These challenges prompt consideration of how data infrastructures can maximise the use of data while protecting the interests of data donors. The UK’s Chief Medical Officer, for example, proposes the creation of ‘data banks’ based on shared expectations and trust between patients, the public, the health service and researchers in the public and private sectors [3]. Here, we consider how data governance arrangements can support ethical and sustainable use of diverse data for research into dementia. We argue that new governance approaches can improve data access, maximising benefit, while operating in accordance with, and protecting, the interests and values of those who donate it.

## Discussion

### Consent and changing data ecosystems

Until recently, data for dementia research have primarily come from clinical records or research data. International data initiatives such as the Dementias Platform UK or the Global Alzheimer’s Association Interactive Network aim to bring these data together and make them widely available. There is also currently growing interest in the potential of digital tools for dementia research, for example, through the use of mobile devices to assess gait, sleep, cognition or speech [2, 4]. While this may result in new forms of data, it involves an imbrication of commercial and public sector research that can be challenging for public trust [5]. Such corporate-clinical collaborations may raise concerns about privacy and corporate use of health data, as in the collaboration between the Royal Free Hospital in London and Google Deepmind [6, 7].

Questions of ethics and governance should be at the heart of how data initiatives develop, to ensure that they can prove their trustworthiness to those who donate and collect data. However, the scope and scale of data for research presents challenges to data governance and ethics. First, it is important to understand whether and how people have consented to the sharing and use of their data. It is also increasingly difficult to ensure that informed consent is meaningful when both future uses and users of data are unpredictable. The value and limits of broad consent, in which consent is provided at the outset for a wide, but not unlimited range of uses, have been the subject of long-standing debate [8].

Dementia research represents a distinctive context for consent, as it may involve vulnerable individuals who may at some point lack the capacity to consent even when supported by family members, carers or researchers. Recommendations for consent processes for data sharing for dementia research developed by Thorogood and colleagues for the Global Alliance for Genomics and Health emphasise that consent must support decision-making by persons with dementia, protect them from exploitation and promote the common good [9]. Thorogood et al. argue that these goals are best achieved through broad consent that is designed to endure beyond a loss of capacity and that is combined with ongoing oversight.

### Governing data

Mechanisms for oversight or governance have received little attention among the dementia research community [10, 11]. Transparent, proportionate and adaptable oversight can support the sharing and use of data by enabling data donors—whether patients, researchers or companies—to trust that uses and users of data align with their values and interests [12].

Innovation in data collection has been accompanied by that in data governance. One model that may have potential in the context of dementia research is that of the data trust [13, 14]. Data trusts have been identified as potentially valuable for health research, and similar approaches have previously been proposed for the governance of bioresources [15–17]. A trust is a legal way to manage rights in an object for the benefit of another person. The essential characteristic of data trusts is that their structure, governance and operating practices make sharing of data possible in a fair, safe and equitable way [15].

A data trust would act as an independent and sustainable steward of data from diverse sources, rather than adding governance to individual studies or being created de novo alongside each data initiative. It could receive and take responsibility for data from researchers, companies or health systems. In the EU, the General Data Protection Regulation further establishes a right to data access for data subjects, and limited rights to portability, which may enable individuals themselves to access and share data.

A trust with the goal of furthering understanding of ageing and dementia would be co-designed by and reflect the values and preferences of all stakeholders, including people with dementia, families and research participants but also the wider public, charities, researchers and companies. This process could build on existing engagement activities with people with and without dementia, as well as deliberative approaches similar to those previously adopted to incorporate wider patient and community perspectives in the development of biobanks [18–20].

Once operational, the use and sharing of these data would be managed by both expert and lay trustees in line with the values established and codified through these deliberations. A trust would commit to making these data available and interoperable, contributing to releasing data held in silos in the public and private sectors. Finally, a trust would be able to exert effective stewardship over data, denying or withdrawing access where necessary.

Data trusts are an emerging concept and their implementation requires piloting and experimentation. Lessons may be learned from the experience of bioresources; for example, the Michigan BioTrust, which has fiduciary responsibility for a repository of neonatal dried blood spots, aims to ensure that research is consistent with public and private interests through a combination of a Community Values Advisory Board alongside Scientific and Ethics Advisory Boards [17]. A series of pilots in non-biomedical domains for the UK Open Data Institute have also explored the potential of trust models. They suggest that for stakeholders and the public to have trust in a data trust, it has to be seen to reflect their issues, expectations and perspective on trade-offs; focus on building consensus; and be open, honest and accountable [13].

As an independent, transparent body, incorporating the interests and perspectives of multiple stakeholders, a dementia data trust could provide an architecture for ‘trustworthy’ oversight by linking the long-term use of data to a clear set of values, goals and principles. This would support the validity of broad consent and enable the protection of the interests of research participants/data donors even in circumstances where they may no longer have the capacity to provide informed consent.

## Summary

Large-scale initiatives based on multimodal data from diverse sources are increasing central to dementia research. Facilitating effective and ethical use of data requires systematic attention to scalable and sustainable frameworks for data governance. This includes prospectively considering the potential of different models to facilitate research in the interests of stakeholders. A model that incorporates features of an independent dementia data trust might provide one such approach.

## Data Availability

Not applicable
